# Special Issue “The Role and Mechanism of Hydrogen Sulfide and ROS in Plants”

**DOI:** 10.3390/ijms27020969

**Published:** 2026-01-19

**Authors:** Zhiya Liu, Zhuping Jin, Weibiao Liao

**Affiliations:** 1 College of Horticulture, Gansu Agricultural University, 1 Yinmen Village, Lanzhou 730070, China; lzyaaa1127@163.com; 2 Shanxi Key Laboratory for Research and Development of Regional Plants, School of Life Science, Shanxi University, Taiyuan 030006, China; jinzhuping@sxu.edu.cn

## 1. Introduction

Hydrogen sulfide (H_2_S), recognized as the third gasotransmitter after nitric oxide and carbon monoxide, is pivotal in plant responses to abiotic stresses, including heavy metal toxicity, salinity, drought, and extreme temperatures [1,2]. It functions through multiple mechanisms: directly enhancing stress tolerance via its metabolic pathways and indirectly forming synergistic networks through interactions with other signaling molecules and phytohormones. These coordinated actions ultimately enable plants to better adapt to adverse environmental conditions [1,2].

Reactive oxygen species (ROS) function as essential signaling molecules, allowing plant cells to rapidly perceive and respond to diverse stimuli. They are pivotal in integrating environmental signals, activating stress response networks, and ultimately enhancing plant tolerance against both abiotic and biotic stresses [3]. Recent research has substantially advanced our understanding of ROS signaling, leading to the identification of specific ROS receptors, key regulatory nodes that interconnect ROS with other stress and hormonal pathways, and the novel roles of ROS in mediating communication both within and between cells [3,4].

This Special Issue, entitled “The Role and Mechanisms of Hydrogen Sulfide and ROS in Plants,” presents a collection of original research articles and reviews that collectively explore the diverse molecular mechanisms through which H_2_S and ROS regulate plant growth and development.

Herein, we aim to showcase the principal advances presented in this Special Issue. Against the backdrop of a rapidly evolving field, in-depth studies of H_2_S and ROS signaling have not only illuminated their multifaceted roles in cellular communication but have also set the stage for the next phase of inquiry. We conclude by outlining essential future research trajectories, focused on the interaction of these factors, which are poised to unravel the sophisticated synergistic networks governing plant stress tolerance.

## 2. Overview of the Special Issue Contributions

A study on cucumber’s response to cadmium (Cd) stress revealed a significant interaction between H_2_S and methyl jasmonate (MeJA) in alleviating Cd toxicity (contribution 1). The exogenous application of either H_2_S or MeJA effectively mitigated the Cd-induced growth inhibition in cucumber seedlings. The protective mechanisms involve reducing ROS accumulation, enhancing antioxidant levels (e.g., AsA and GSH) and the expression of related genes, and maintaining photosynthetic system stability by downregulating chlorophyll degradation genes (e.g., *PAO*, *RCCR*, *NYC1*). Crucially, the positive effects of H_2_S were reversed by a MeJA synthesis inhibitor, positioning MeJA as a downstream component in the H_2_S signaling pathway (contribution 1). These findings elucidate a synergistic mechanism between H_2_S and MeJA in Cd stress tolerance, offering new theoretical insights for plant stress resistance.

Wang et al. elucidated the synergistic interaction between H_2_S and abscisic acid (ABA) in mediating thermotolerance in maize seedlings under heat stress(contribution 2). Their findings demonstrate that H_2_S and ABA reciprocally upregulate each other’s endogenous levels through the modulation of key metabolic enzymes and associated gene expression. Exogenous application of either H_2_S and ABA, as well as their combined treatment, significantly enhanced seedling survival, mitigated membrane damage, and attenuated oxidative stress. Mechanistically, the H_2_S-ABA crosstalk activated the reactive oxygen species (ROS) scavenging system, encompassing both enzymatic antioxidants (e.g., catalase, CAT; ascorbate peroxidase, APX; superoxide dismutase, SOD) and non-enzymatic antioxidants (e.g., ascorbate, AsA; carotenoids). Consequently, this interaction reduced heat-induced ROS accumulation and substantially improved plant thermotolerance (contribution 2). These results provide novel insights into the coordinated roles of H_2_S and ABA signaling in enhancing plant resilience to high-temperature stress.

In plants, respiratory burst oxidase homologs (RBOHs) serve as key enzymes catalyzing the production of ROS, and play central regulatory roles in growth, development, and stress adaptation. Jiang et al. systematically identified and characterized the NADPH oxidase (RBOH) gene family in oat (*Avena sativa*) at the whole-genome level (contribution 3). A total of 35 *AsRBOH* genes were identified, distributed across 14 chromosomes, and phylogenetically classified into five distinct subfamilies. Promoter *cis*-element analysis indicated the presence of multiple stress- and hormone-related motifs, including those responsive to drought, hypoxia, and phytohormones. Expression profiling under abiotic stress revealed that 28 *AsRBOH* genes were upregulated under drought conditions, whereas 18 were downregulated under salt stress. Additionally, several genes displayed diurnal expression patterns during different seed developmental stages(contribution 3). These results establish a genomic and transcriptomic foundation for further functional studies on the *RBOH* gene family in oat growth, development, and stress responses.

Wang et al. conducted a systematic genome-wide identification and functional analysis of the GROWTH-REGULATING FACTOR (GRF) gene family in *Populus euphratica* Oliv., a species exhibiting leaf heteromorphism (contribution 4). Their study identified *PeGRF9* as a key regulator associated with leaf morphology. Transcriptomic analysis (RNA-seq) combined with qRT-PCR validation revealed that *PeGRF9* expression was significantly elevated in broad leaves compared to narrow leaves. Functional characterization through heterologous overexpression in *Arabidopsis thaliana* demonstrated that *PeGRF9* could substantially increase leaf area(contribution 4). These findings establish a crucial molecular link between *PeGRF9* expression and leaf expansion, thereby providing a theoretical foundation for elucidating the genetic mechanisms governing heteromorphic leaf development in *P*. *euphratica*.

To evaluate the effects of nanoparticle-enhanced fertilization on tomato plants, Włodarczyk et al. investigated the regulatory effects of zinc oxide nanoparticles (nano-ZnO), applied either alone or in combination with conventional fertilizers, on plant growth, development, and the antioxidant system(contribution 5). The study systematically examined the influence of concentration, application method (foliar spray vs. soil application), and cultivar specificity on tomato antioxidant capacity. Results indicated that the impact of nano-ZnO was highly dependent on these factors. Foliar spraying, in certain contexts, proved more effective than soil application, particularly in enhancing specific antioxidant enzyme activities and non-enzymatic antioxidant levels. An optimal concentration range (e.g., 50-150 mg/L) was found to strengthen the plant’s antioxidant defense system and improve its adaptability to environmental stress (contribution 5). The study concludes that nano-ZnO holds promise as a nano-fertilizer, but its effective use requires precise optimization of concentration and application strategy, taking into account cultivar-specific responses.

Emerging research indicates that H_2_S interacts with the ROS-mediated oxidative stress network through complex, multi-level mechanisms [5]. This paradigm shifts the traditional view of H_2_S as a mere ROS scavenger, revealing its multifaceted regulatory roles in redox homeostasis. A comprehensive review by Liu et al. (contribution 6), entitled “Hydrogen Sulfide in the Oxidative Stress Response of Plants: Crosstalk with Reactive Oxygen Species” systematically elucidates the molecular underpinnings of H_2_S-ROS crosstalk. Key mechanisms highlighted include (i) the direct reaction of H_2_S with oxidized cysteine residues (e.g., sulfenylation) on target proteins, and (ii) the critical role of ROS as both upstream inducers and downstream mediators in H_2_S signaling pathways. The review further underscores the significant synergistic effects between H_2_S and ROS in fine-tuning cellular redox balance, emphasizing their collaborative biological significance in plant adaptation to abiotic and biotic stresses.

## 3. Conclusions

The six articles in this Special Issue collectively advance our understanding of H_2_S and ROS signaling in plants, spanning from gene discovery in non-model species to the functional characterization of key regulators, and further elucidate their crosstalk with other signaling pathways under abiotic stress. Together, they systematically map a synergistic H_2_S-ROS regulatory network governing critical plant physiological processes. These findings present significant opportunities for agricultural innovation, particularly in enhancing crop stress resilience, optimizing seed systems, and ultimately improving productivity. At the molecular level, the studies clarify the essential biological role of H_2_S-ROS synergy in plant stress adaptation. This mechanistic insight provides a robust theoretical foundation for designing future strategies in precision agriculture and targeted crop improvement. These groundbreaking contributions not only deepen fundamental knowledge of plant redox signaling networks but also open novel research avenues for addressing pressing global challenges, such as climate change and food security, thereby displaying substantial scientific and practical relevance.
